# Artificial intelligence and the analysis of multi-platform metabolomics data for the detection of intrauterine growth restriction

**DOI:** 10.1371/journal.pone.0214121

**Published:** 2019-04-18

**Authors:** Ray Oliver Bahado-Singh, Ali Yilmaz, Halil Bisgin, Onur Turkoglu, Praveen Kumar, Eric Sherman, Andrew Mrazik, Anthony Odibo, Stewart F. Graham

**Affiliations:** 1 Department of Obstetrics and Gynecology, William Beaumont Health, Royal Oak, MI, United States of America; 2 Oakland University-William Beaumont School of Medicine, Rochester, MI, United States of America; 3 Department of Computer Science, Engineering and Physics, University of Michigan-Flint, Flint, MI, United States of America; 4 University of Michigan, Ann Arbor, MI, United States of America; 5 Department of Obstetrics and Gynecology, University of South Florida, Tampa, FL, United States of America; Hopital Robert Debre, FRANCE

## Abstract

**Objective:**

To interrogate the pathogenesis of intrauterine growth restriction (IUGR) and apply Artificial Intelligence (AI) techniques to multi-platform i.e. nuclear magnetic resonance (NMR) spectroscopy and mass spectrometry (MS) based metabolomic analysis for the prediction of IUGR.

**Materials and methods:**

MS and NMR based metabolomic analysis were performed on cord blood serum from 40 IUGR (birth weight < 10^th^ percentile) cases and 40 controls. Three variable selection algorithms namely: Correlation-based feature selection (CFS), Partial least squares regression (PLS) and Learning Vector Quantization (LVQ) were tested for their diagnostic performance. For each selected set of metabolites and the panel consists of metabolites common in three selection algorithms so-called overlapping set (OL), support vector machine (SVM) models were developed for which parameter selection was performed busing 10-fold cross validations. Area under the receiver operating characteristics curve (AUC), sensitivity and specificity values were calculated for IUGR diagnosis. Metabolite set enrichment analysis (MSEA) was performed to identify which metabolic pathways were perturbed as a direct result of IUGR in cord blood serum.

**Results:**

All selected metabolites and their overlapping set achieved statistically significant accuracies in the range of 0.78–0.82 for their optimized SVM models. The model utilizing all metabolites in the dataset had an AUC = 0.91 with a sensitivity of 0.83 and specificity equal to 0.80. CFS and OL (Creatinine, C2, C4, lysoPC.a.C16.1, lysoPC.a.C20.3, lysoPC.a.C28.1, PC.aa.C24.0) showed the highest performance with sensitivity (0.87) and specificity (0.87), respectively. MSEA revealed significantly altered metabolic pathways in IUGR cases. Dysregulated pathways include: beta oxidation of very long fatty acids, oxidation of branched chain fatty acids, phospholipid biosynthesis, lysine degradation, urea cycle and fatty acid metabolism.

**Conclusion:**

A systematically selected panel of metabolites was shown to accurately detect IUGR in newborn cord blood serum. Significant disturbance of hepatic function and energy generating pathways were found in IUGR cases.

## Introduction

Fetal growth restriction (FGR) or alternatively Intrauterine growth restriction (IUGR) refers to inadequate fetal growth due to pathological reasons [1]. This is a common pregnancy complication. In the U.S.A. the diagnosis is based on an estimated fetal weight (EFW) less than the 10th percentile for gestational age. While other prenatal assessments are frequently used, for example Doppler, amniotic fluid volume, or a small abdominal circumference, they are currently not required to make the diagnosis. Ultimately, the gold standard is confirmation of a birth weight less that the 10th percentile for gender, ethnicity, and gestational age, termed small for gestational age (SGA). IUGR is associated with increased risk of stillbirth, and perinatal morbidity and mortality [2]. The increased risk extends into childhood and adulthood and include obesity and vascular disorders such as coronary artery disease [3].

A significant percentage of fetuses and newborns with an estimated fetal weight (EFW) or birthweight below the 10th percentile is small for non-pathological or constitutional reasons which is not associated with increased adverse outcomes. Neither current prenatal evaluation techniques nor birthweight percentile adequately distinguish pathological from constitutional small stature. False positive diagnosis i.e. mistaking constitutional small stature for IUGR may be associated with a host of negative consequences including unnecessary fetal testing, and pregnancy interventions that could culminate in iatrogenic prematurity. Current tests for IUGR determination lack sufficient diagnostic accuracy.

Metabolomics has the potential to both generate novel biomarkers for IUGR, distinguish constitutional small stature from IUGR, and provide new insights into its pathogenesis. While there are limited data on metabolomic profiling in IUGR, small available studies suggest excellent accuracy for discriminating IUGR from appropriate for gestational age fetuses [4]. In addition, information on the metabolic disturbance involved in IUGR [5] has been generated.

Machine learning or artificial intelligence (AI) refers to the ability of computers to ‘learn’ from past experience and apply lessons learned to new data-set without the need for prior explicit programming. Artificial Intelligence (AI) models therefore emulate human learning and decision-making [6] Thus, from a prior data-set of 'experience' accurate classifications and predictions can be made [7] Specifically, the computer programs are able to generate models that predict the likelihood of given outcomes and identifies and clarifies contributory features associated with or that gives rise to the outcome of interest and hence pathogenic data.

The unprecedented advances in metabolomic instrumentation accompanied by the generation of large volumes of data requires the parallel development of state of the art analytic techniques [8].

Machine learning techniques are better able to handle the high volume of data generated from a relatively limited number of subjects commonly encountered in systems biology, for example genomic sequencing. The approach improves identification of important features of a data set, model performance and the understanding of the significance of such data compared to traditional analytic approaches [9].

Preliminary results suggest high accuracy for biomarker prediction of disease states and improved insights into disease biology [8].

There has been limited utilization of AI to analyze genomic data. So far however there are very few studies that use machine learning techniques for analysis of metabolomic data. An integrated analytic method using NMR-based blood metabolomics and least squares-support vector machine reported high predictive accuracy for the detection of major depression [10]. An area under the receiver operating characteristics curve (ROC) of 0.96 was achieved for depression prediction. Deep neural network, a form of machine learning was found to be significantly superior to multivariate classification methods such as principal component analysis (PCA) and partial least squares (PLS) methods that are currently routinely used [11] Using a novel approach we combined ^1^H NMR and direct injection coupled with liquid chromatography tandem MS (DI-LC-MS/MS) based metabolomic analyses along with multiple machine learning approaches to predict IUGR and to help elucidate the biology of this disorder. Fetal tissue, represented by cord blood obtained immediately after birth, was used for analysis.

## Material and methods

### Study population and sample collection

A total of 40 pregnant women with suspected IUGR, confirmed at the time of delivery with a birthweight <10^th^ percentile for the appropriate gestational age and 40 maternal age-matched controls with uncomplicated term pregnancies were included in this study (S1 Table). Institutional Review Board (IRB) approval was provided by William Beaumont Hospital (approval #2015–136). Following written consent, venous cord blood samples were collected within the 20 minutes of delivery before the placenta was delivered. At the time of sample collection, venous blood gasses were analyzed from each sample and were found to be with normal pH range suggesting no acute fetal metabolic or respiratory acidosis. Blood samples were centrifuged at 3,000 *g* and serum was aliquoted (0.5 ml) and stored at -80°C freezer within 1 hour. Complicated pregnancies with multiple fetuses and congenital anomalies were excluded from the study. The percentile of growth for each IUGR case and control was confirmed after the delivery with the assessment of infant’s birth weight. Further, we performed subgroup analysis on fetuses with birth weight <5^th^ %ile. There were15 cases (<5^th^ % ile) and 65 controls (≥5^th^ % ile).

### ^1^H NMR sample preparation and data acquisition

Serum specimens were prepared as described by Mercier et al., (2011) [12]. In brief, in order to remove any residual glycerol 3 KDa cut-off centrifugal filter units (Amicon Microcon YM-3; Sigma-Aldrich, St. Louis, MO) were seven times rinsed through centrifugation (12,000 *g* for 30 min) using 0.5 ml of H_2_O. Subsequently, 250 μl of serum was transferred to the filter units and centrifuged at 13,000 *g* for 30 min at 4°C. 200 μl of filtered serum was mixed with 25 μl of D_2_O and 21 μl of a standard buffer solution consisting of 11.7 mM DSS [disodium-2,2-dimethyl-2-silapentane-5-sulphonate], 1.75 M K_2_HPO_4_, and 5.84 mM 2-chloro pyrimidine-5-carboxylic acid (phasing standard) in H_2_O. Using an Eppendorf liquid handler, 200 μl of the final solution were transferred to 3 mm NMR tubes for NMR data collection.

All ^1^H-NMR metabolomics data were collected on a Bruker Avance III HD 600 MHz spectrometer coupled with a 5 mm TCI cryoprobe (Bruker-Biospin, Billerica, MA, USA) at 300.0 K (±0.05). All 1D ^1^H NMR spectra were randomly acquired using a pulse sequence developed by Ravanbakhsh et al.[13]. Two hundred and fifty-six transients were acquired. Chemical shifts are reported in parts per million (ppm) of the operating frequency. DSS was chosen as the internal standard for chemical shift calibration and metabolite quantification All collected spectra were analyzed using a custom library of 58 metabolites using Bayesil [13].

### Combined Direct Injection and LC-MS/MS (DI-MS) compound identification and quantification

A comprehensive description of this analysis has been described by our group previously [14]. Briefly, an AbsoluteIDQ p180 kit (Biocrates Life Sciences AG, Innsbruck, Austria) was used on a TQ-S mass spectrometer coupled to an Acquity I Class ultra-pressure liquid chromatography (UPLC) system (Waters Technologies Corporation, Milford, MA, USA) for targeted analysis. The system provides the accurate quantification of up to 180 endogenous metabolites consisting amino acids, acylcarnitines, biogenic amines, glycerophospholipids, sphingolipids, and sugars. Serum samples were analyzed using the protocol described in AboluteIDQ manual.

### Data processing

**Fig 1:**
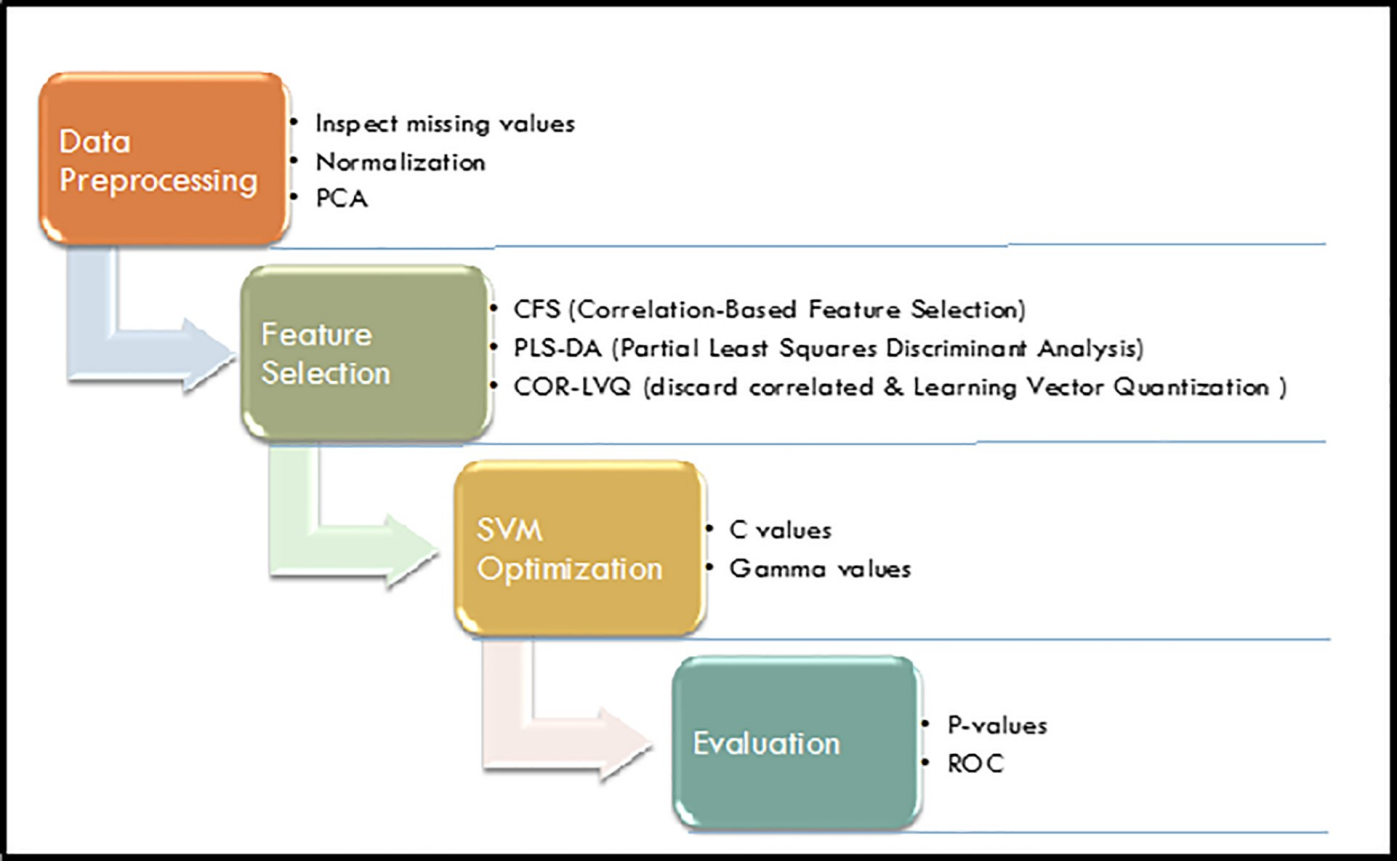
Study design from data preprocessing through performance evaluation.

The workflow for data processing as depicted in Fig 1 was used to develop inclusion/exclusion criteria for variables with missing values or concentration values below the limit of detection.

A metabolite was conservatively excluded if it had missing data in >50% of each group. For all other metabolites, missing measurements were imputed with zero filing. To account for the variation due to dilution effect sum-to-one normalization for each sample was applied, which was followed by z-score normalization for each metabolite. This ensures all values were mapped to a standard scale safeguard compatibility. Finally, principal component analysis (PCA) was performed on the normalized data to identify any potential outliers.

### Feature selection

We performed feature selection analysis on the two groups of cases and corresponding controls: birthweight (<10^th^ ile and <5^th^ %ile), to develop statistical models for prediction of IUGR. To reduce the number of metabolites being used to develop the predictive model, a variety of tools were employed, each offering a different statistical approach. In doing so, we aimed to find the most informative fifteen features (metabolites) and to determine if a consensus exists between the different feature selection algorithms. Initially, we applied learning vector quantization (LVQ) after discarding highly correlated metabolites using the CARET package in R [15]. Secondly, we employed a correlation-based feature selection (CFS) algorithm [16]-a WEKA [17] tool. As for the third approach, we used Partial Least Squares Discriminant Analysis (PLS-DA) to identify the top fifteen important metabolites capable of distinguishing IUGR cases from controls. We then compared the resulting metabolite sets from each method to determine if there is a common set or a consensus that warrants further investigation.

### Incorporating clinical information

Maternal race, age, medical disorder, gravidity, and prior IUGR made up the clinical data available. These were mapped to the same scale as the metabolomic data and we evaluated their performance in combination with the optimal metabolite panel.

### Support vector machines and parameter optimization

Support Vector Machine (SVMs) classification is a machine-learning algorithm, prominent for its robustness and capability in handling both linear and non-linear data. In this study, we adopted a radial basis function (RBF) as the kernel function which is more practical [18]. However, both RBF and the SVMs parameters were identified following an exhaustive search. This included finding the best *γ* for RBF in Eq 1 where *x* and *y* represent feature vectors for two data points.

$$K(x,y)=exp(\gamma {\left\|x-y\right\|}^{2})$$ (1)

Additionally, we needed to determine the optimum *C* value, which is the penalty parameter in the SVM formulation.

In this current work, we used Scikit-learn [19], a machine-learning library in Python and performed our exhaustive search to obtain the best *C*-*γ* pair on a grid that was laid on exponentially varying *C* and *γ* values, i.e., *C* ∈ [e+01, to e+5] and *γ* ∈ [e-01, e-06]. More specifically, we employed a 10-fold cross validation for all thirty *C*-*γ* combinations, aiming for the highest accuracy, which is the ratio of truly predicted samples as described in Eq 2 below.

$$accuracy=\frac{TP+TN}{TP+TN+FP+FN}$$ (2)

### Performance evaluation for SVM models

To assess the performance and the significance of the optimized the model, we performed a permutation test (1000 iterations). Permutation testing basically shuffles the class labels and uses the same set of metabolites in the 10-fold cross validation. Distribution of prediction accuracies were used to assess the significance of the optimized model. As the final step, we calculated true/false positives and true/false negatives for each round of our 10-fold cross validation to report average sensitivity (TP/P) and specificity (TN/N) values along with the average of the area under the curve (AUC).

### Metabolite set enrichment and network analysis

Using metabolite set enrichment analysis (MSEA;MetaboAnalyst (v 3.0) [20] we analyzed the raw data to identify any metabolic pathways which may have been significantly perturbed due to IUGR. In this workflow the *Homo sapiens* (human) pathway library was chosen and all of the compounds in the selected pathways were used when referencing the specific metabolome. MSEA directly examines if a group of functionally related metabolites are considerably enriched, with no preselection of compounds based on some arbitrary cut-off value. Potentially, it can identify "subtle but consistent" changes among a group of related compounds, which may go undetected using conventional approaches [20]. Multiple comparisons were carried out using the more stringent Holm adjusted p-values.

## Results

A total of 40 IUGR cases (<10^th^ %ile) and 40 maternal age-matched controls (≥10^th^ %ile) with uncomplicated pregnancies were included in the study which is reduced to be 39 cases and 39 controls after outlier detection. We accurately identified and quantified 58 metabolites using ^1^H NMR and 180 metabolites using DI-LC-MS/MS. Some overlap was observed across the two platforms when measuring metabolite concentrations (n = 24) and as a result we took the average of both measurements for our analyses. Not all recorded metabolites were found to be above the LOD and as such we ended up using a total of 207 metabolites. Using PCA we identified one subject from each group to lie outside the 95^th^ percentile and excluded them from further analyses (S1 Fig).

**Fig 2:**
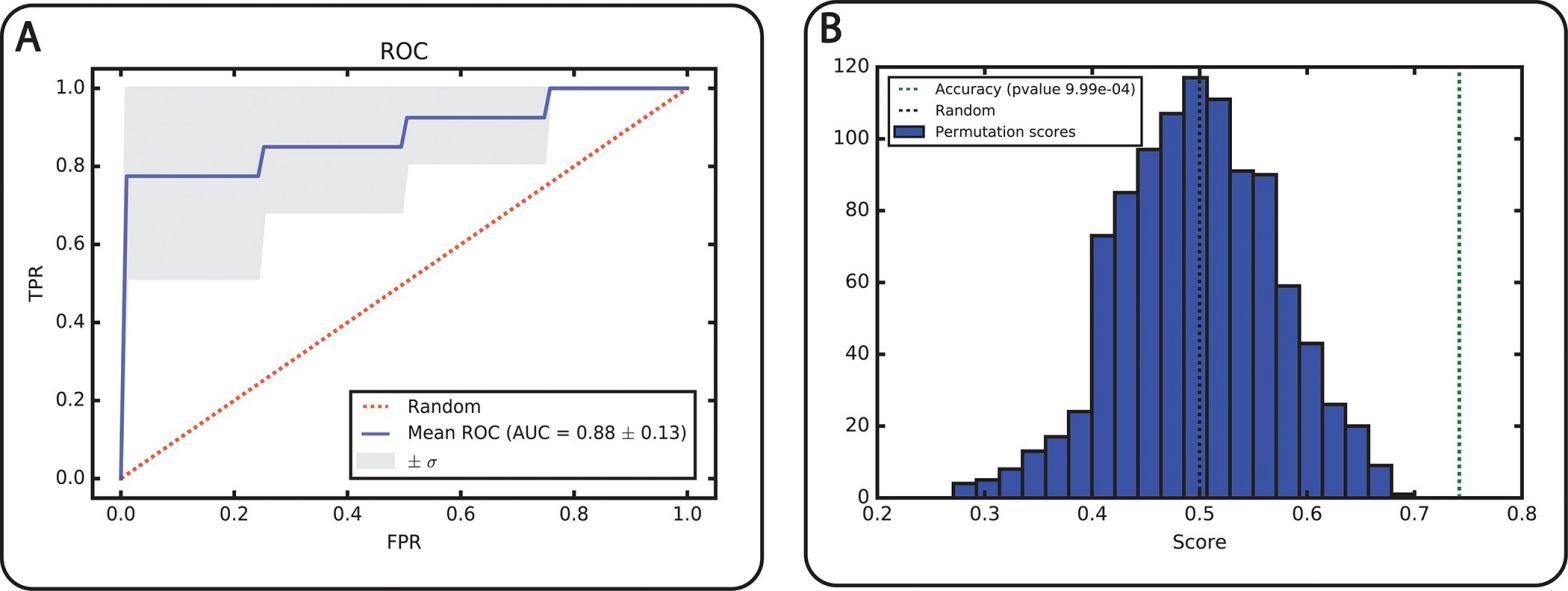
A) ROC with AUC values, B) permutation test obtained from the OL set.

Table 1. represents the metabolite panels sorted in descending order of importance for each feature selection method used i.e., CFS, PLS, and COR-LVQ. The last column lists the overlapping (OL) metabolites identified as being important in all three methods. For each feature set, we performed a 10-fold cross validation to optimize *C*-*γ* pairs among which we report the best pairs (S2 Table). Among the three methods, the CFS and PLS features led to better average accuracy rates (0.80) with standard deviations of 0.15 and 0.14, respectively. While the entire metabolite set was able to reach an accuracy of 0.77, the OL set of metabolites demonstrated the best performance in terms of average accuracy. Regardless of the choice of feature set, model accuracies were found be to be statistically significant (*p*<0.05) using permutation testing (Fig 2A).

**Table 1:** Selected metabolites as identified using CFS, PLS-DA, COR-LVQ and their common compounds.

Feature selection schemes				
**Metabolite Panel**	**CFS**	**PLS**	**COR-LVQ**	**OL**
	Creatinine	lysoPC.a.C16:1	Creatinine	Creatinine
	C14	C2	lysoPC.a.C16:1	C2
	C2	Creatinine	lysoPC.a.C20:3	C4
	C4	lysoPC.a.C18:2	C2	lysoPC.a.C16:1
	lysoPC.a.C16:1	lysoPC.a.C18:1	lysoPC.a.C18:2	lysoPC.a.C2:.3
	lysoPC.a.C18:1	lysoPC.a.C20:3	C4	lysoPC.a.C28:1
	lysoPC.a.C20:3	PC.aa.C24:0	C12:1	PC.aa.C24:0
	lysoPC.a.C20:4	C6.C4:1.DC.	EDTAca_N	
	lysoPC.a.C28:1	C4	C6.C4:1.DC.	
	PC.aa.C24:0	C10:1	Taurine	
	PC.aa.C36:4	C16:1	C16:2	
	PC.aa.C38:4	C12:1	C0	
	PC.aa.C42:4	C12	Putrescine	
	EDTAca_N	C0	PC.aa.C24:0	
	Creatine	lysoPC.a.C28:1	lysoPC.a.C28:1	

CFS: correlation-based feature selection, PLS: Partial least squares regression. COR-LVQ: Correlation based Learning Vector Quantization earning. O: Overlapped panel

**Fig 3:**
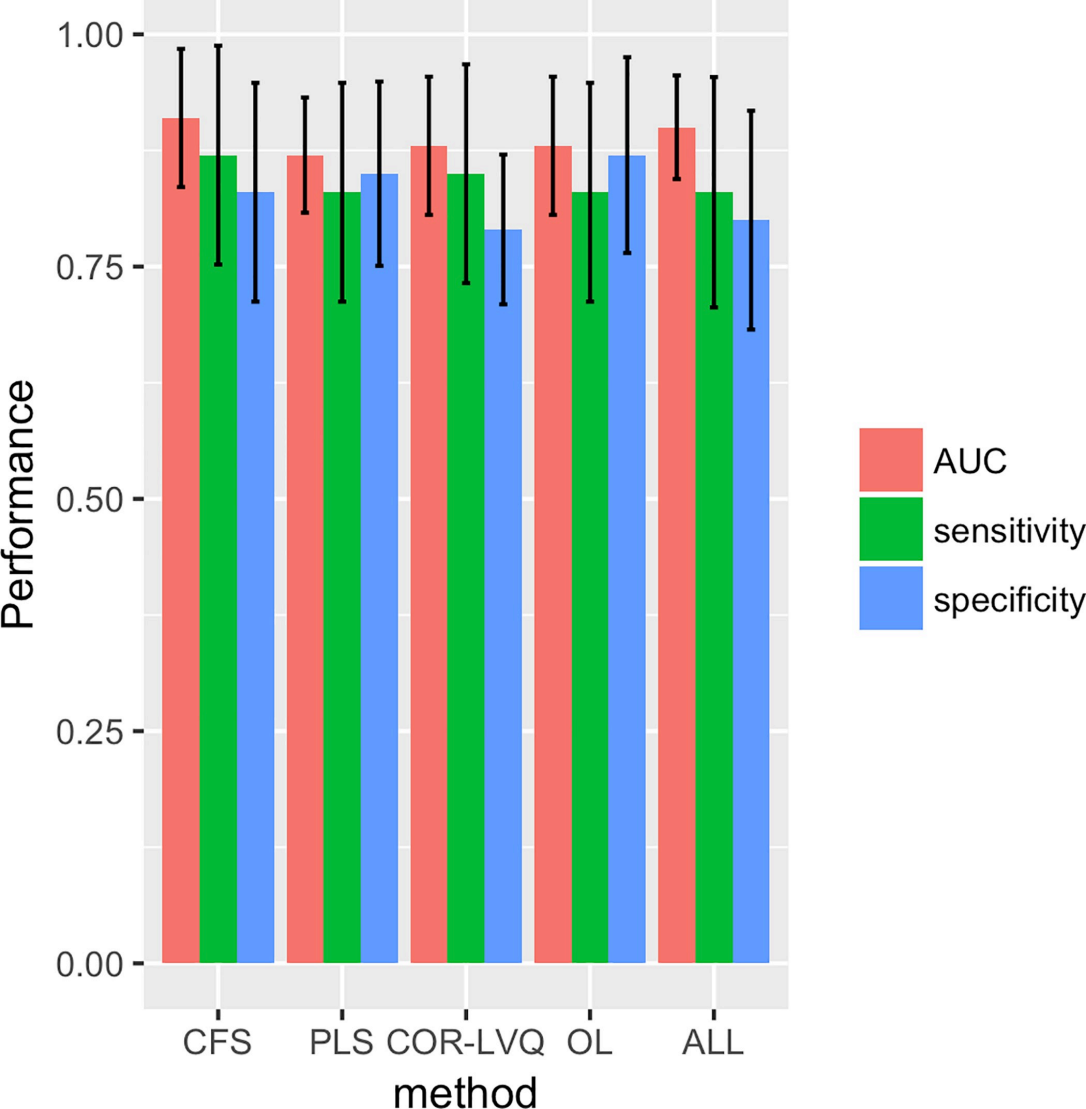
Performance evaluation in terms of sensitivity, specificity, and AUC for all metabolite panels obtained different variable selection algorithms.

Sensitivity and specificity values were calculated for all models and are listed in S2 Table. Using a 10-fold cross validation method we report each model evaluation as averages and standard deviations (n = 10 rounds). In Fig 2B we present a ROC curve for the common set of metabolites which has an AUC = 0.88 with σ = 0.12. We summarized the performance measurements for all variable selection methods in Fig 3 (average AUC = 0.87–0.91). We found that the CFS selected metabolite panel has the highest AUC (= 0.90) with sensitivity = 0.87 and specificity = 0.83. However, the OL panel demonstrates the best performance in terms of the average specificity, thus demonstrating its capacity to correctly classify negative instances with low FP rate. While the COR-LVQ had the lowest average specificity = 0.79, the model based on all metabolites did not outperform PLS, CFS, nor OL Further, we found that the variance observed for each of the average AUC values is substantially less than that of the associated specificity and sensitivity values.

Finally, we incorporated all the available clinical data into our model to include prior history of IUGR, maternal age, gravity and race and evaluated the prediction performance of the model using a 10-fold cross validation. A combination of the OL metabolites and the clinical information did not perform as well as the metabolite only model with an AUC = 0.64 (high standard deviation = 0.21). Clinical data alone produced an AUC = 0.54 (p = 1.0). These results indicated that metabolites by themselves performed better than some standard clinical risk factors for predicting IUGR.

In addition to analysis above, we used the same approach to predict severe IUGR cases <5^th^ ile. We were able to develop highly accurate models. The performance metrics of each statistical model and selected panel of metabolites based on OL modeling can be found in S3 Table. We found that the PLS and COR-LVQ selected metabolite panels achieved the highest accuracy with: AUC = 0.90. Using the CFS panel a sensitivity of 1.0 and specificity of 0.88 was achieved. The COR-LVQ selected metabolite panel yielded the highest specificity = 0.93 with a sensitivity of 0.92.

### Metabolite set enrichment and network analysis

**Fig 4:**
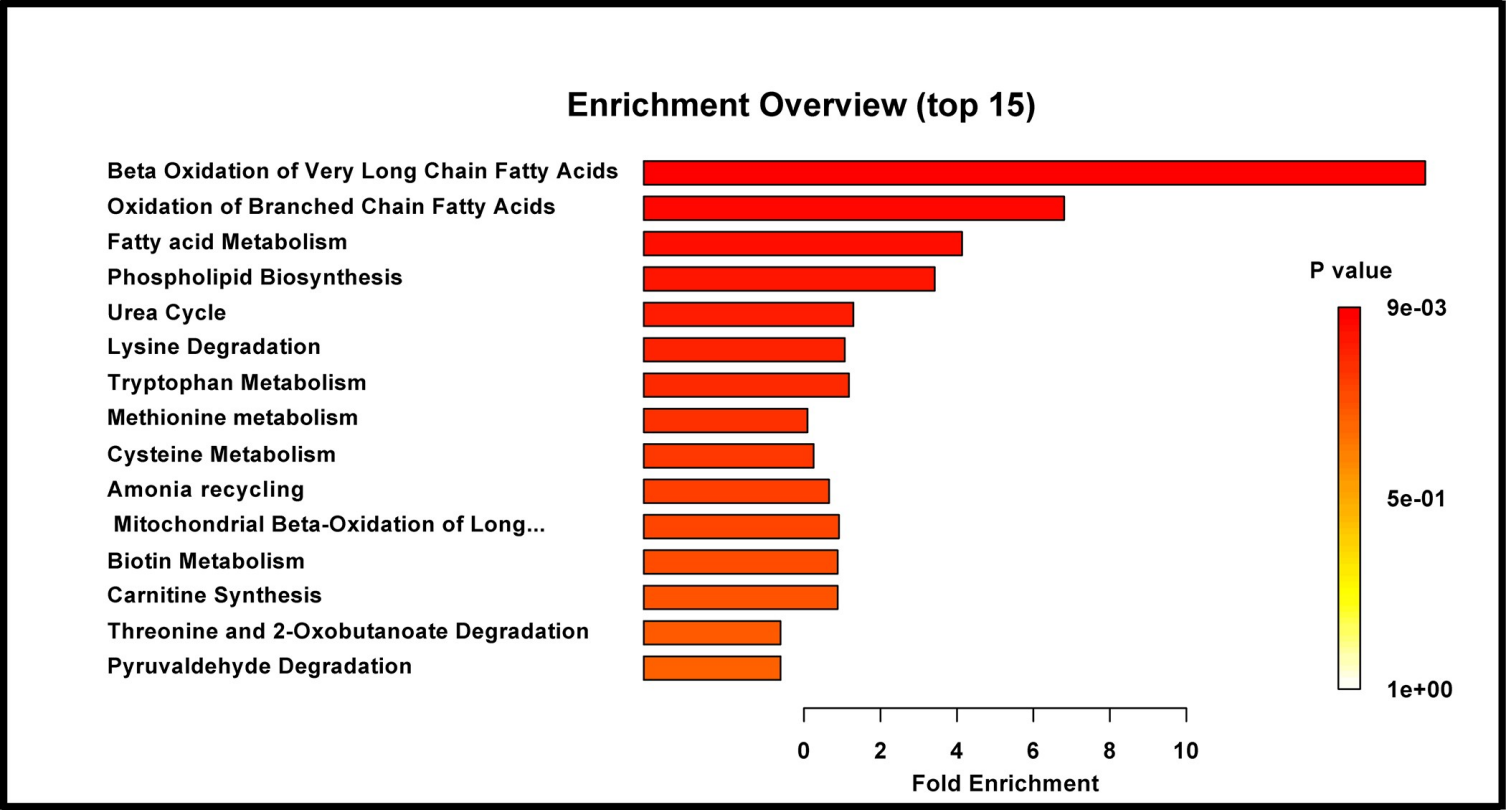
Bar graph: altered pathways in enrichment analysis showing top fifteen metabolic pathways perturbed upon IUGR.

Metabolite set enrichment analysis (MSEA) showed multiple metabolic pathways that were significantly dysregulated in IUGR cord blood (p-<0.05). Table 2 presents each perturbed biochemical pathway with number of metabolite hits, p-values, holm-adjusted p-values and false discovery rates. The column “total” represents the total number of metabolites involved in a particular metabolic pathway. The column “hits” indicates the number of metabolites in that particular pathway found to have significant concentration changes in the cord blood. Pathways with the highest number of hits and a significant Holm p-values are the most significantly perturbed in IUGR. Fig 4 highlights the fold enrichment which is obtained when using the raw concentration data over conventional metabolic pathway analysis. The color intensity correlates with the level of statistical significance of each pathway while the length of each bar represents the fold enrichment of each pathway when cases are compared to controls based on the quantitative concentration data. The p-values of pathways were determined by the difference of concentration data and the number of participating metabolites. Fig 4 thus visually portrays the metabolic pathways that were disproportionately affected (over-represented). The top three most significant pathways included: beta oxidation of very long chain fatty acids, oxidation of branched chain fatty acids and phospholipid metabolism. In addition, lysine degradation, urea cycle, phospholipid biosynthesis, tryptophan metabolism, and fatty acid metabolism were also significantly perturbed.

**Table 2:** Pathway analysis of intrauterine growth restriction.

Metabolite Set	Total^+^	Hits^	P value	Holm P*	FDR
Beta oxidation and very long fatty acids metabolism	14	4	0.00007	0.0034	0.0034
Oxidation of branched chain fatty acids metabolism	14	3	0.00032	0.0014	0.0079
Fatty acid metabolism	19	2	0.012	0.044	0.0155
Phospholipid biosynthesis	13	4	0.018	0.002	0.0245
Urea cycle	15	3	0.032	0.04	0.0493
Methionine Metabolism	24	8	0.042	0.046	0.0490
Lysine degradation	18	1	0.046	0.05	0.0550
Tryptophan metabolism	34	2	0.049	0.05	0.0630

^+^ Total number of metabolites in given pathway

^ Number of significant metabolites (p<0.05) (FGR vs controls) in given pathway

* Holm-Bonferroni Method is used

## Discussion

We evaluated for the first time the combined use of ^1^H NMR and DI-LC-MS/MS for biochemical profiling of cord blood serum, representing fetal metabolism, from IUGR patients and compared them with controls. We performed our analysis based on birth weight <10^th^ ile which is the current clinical criteria for the management of IUGR. However, in order to validate the strength of our metabolite modeling, we also performed subgroup analysis for severe IUGR (birth weight <5^th^ ile) which were also supported the value of our predictive modeling. Briefly, we have exhaustively evaluated numerous variable selection techniques to identify the most robust panel of potential metabolite biomarkers for the detection of IUGR in cord blood serum using support vector machine learning algorithms. Previous studies have attempted to do this but have been limited in their approach (using ^1^H NMR or tandem MS alone) [21, 22]. To identify the most informative and robust metabolite panel for the confirmation of IUGR at birth, we began with a comparative approach which used all recorded data/metabolite concentrations. We performed a 10-fold CV to optimize the SVM parameters where accuracy or true prediction ratio was the single criterion. Our parameter space was logarithmically designed and exhaustively visited to seek the best accuracy. This is common practice when optimizing SVM models.

We performed SVM immediately after the preprocessing step and found that the complete data set produced an average diagnostic accuracy of 77% (σ = 0.16) following a 10-fold CV. In the subsequent experiments, we employed three different feature selection methods and used the top 15 metabolites from each. We then noticed that even the lowest performing approach, COR-LVQ, was able to reach 78% with a subset of only 15 features, which still outperformed the model that utilized whole feature set.

In terms of AUC, the average AUC we got through CFS features was 0.91, which was the highest performing of all the analytic approaches and showed the utility of a subset over all available metabolites. We further observed the prediction power of those subsets of features obtained through different approaches despite their slightly lower AUC values.

In this study we wanted to use metabolites which were common to all variable selection methods to produce the most robust diagnostic algorithm. We found creatinine, acetyl carnitine (C2), butyryl carnitine (C4), three lysophosphatidylcholines (lysoPC.a.C16.1, lysoPC.a.C20.3 and lysoPC.a.C28.1) and a phosphatidylcholine (PC.aa.C24.0) to overlap across all selection techniques and used these to develop our diagnostic algorithm. We repeated the CV steps for optimizing the SVM parameters (average diagnostic accuracy = 88%, sensitivity = 0.83 and specificity = 87) and demonstrate that this panel was the most accurate for diagnosing IUGR in cord blood serum. By performing additional variable selection techniques, we believe that we have developed the most robust and accurate biomarker panel for IUGR diagnosis using metabolomics-based measurements in cord blood. One implication of this panel is that we actually achieved the goal of reducing the number of potential biomarkers, which is definitely a cost-effective diagnostic approach in a hospital environment. It is also important to note that while we evaluated two metabolite platforms metabolites measured using DI-LC-MS/MS were found to be the most useful when developing the final diagnostic panel. The need for only a single platform would be economically beneficial for clinical testing.

We incorporated standard clinical and demographic information in order to boost the performance of our biomarker panel. The inclusion of these data however decreased the power of our diagnostic model emphasizing that biochemical markers alone were independent and accurate predictors of IUGR. While cord blood is not available in most clinical contexts, it is possible that these panel of biomarkers identified in the cord blood serum, may also be present in the maternal blood. This is because of the small molecular weights of metabolites and easy diffusability into other fluid compartments such as maternal blood. The development of similar markers in maternal blood would aid prenatal diagnosis and interventions that could minimize perinatal morbidities.

A majority of small for gestational age newborns, have constitutional small stature not associated with increased perinatal morbidities. In contrast, pathological growth restriction which increases the risk of perinatal morbidities and even death is often indistinguishable from constitutional causes of small birth weight. There is significant scientific interest in determining the cellular mechanisms of small size in the fetal period and at birth and in distinguishing pathological from benign causes of poor fetal growth. Metabolomic analysis has the capability of interrogating the cellular mechanisms and thus the potential pathogenesis of IUGR. Fig 4 visually portrays the metabolic pathways that were disproportionately affected (over-represented) or perturbed. The fetal liver plays a central role in many of these metabolic activities e.g. bile acid, fatty acid synthesis metabolism. The cord blood analysis appears to indicate abnormal fetal liver function. Significant changes in the liver reflected in small liver size and abdominal circumference is a well-accepted feature of in-utero growth restriction [23]. Interestingly, significant changes in phosphotidylcholines concentrations in cord blood have been previously reported to be (S4 Table) positively correlated with birth weight [21]. Abnormal lipid metabolism could be explained by chronic hypoxia and reduced levels of placental energy substrates. Moreover, hypoxia can cause significant changes in lipid metabolism [24]. Metabolite set enrichment analysis revealed perturbations in beta oxidation of very long chain fatty acids and oxidation of branched fatty acids pathways (Table 2). Bartha and colleagues reported (2012) significantly reduced levels of long-chain 3-hydroxyacyl-CoA dehydrogenase and fatty acid oxidation capacity in placentas from women with preeclampsia, a disorder that is commonly associated with growth restriction [25]. However, in contrast to what we observed perturbations in beta oxidation of very long chain fatty acid would be expected to result in the accumulation of long chain acylcarnitine with a relative decrease or absence of the short chain acylcarnitine upon perturbation of beta oxidation of very long chain fatty. Metabolites are known to be in a state of active flux and many pathways affect the levels of individual metabolites. The elevation of short chain acetylcarnitines (C2, C4) is due to increased turnover of TCA metabolites and their usage for gluconeogenesis, which leads to a decrease in oxaloacetate levels and accumulation of acetyl-coA. Increased acetyl-coA leads to increased levels of acetylcarnitines and ketone bodies such as 3-hydroxybutyric acid and acetoacetate (S4 Table) as was found in our findings. Hence, the contradictory findings of decreased long chain fatty oxidation and elevated levels of short chain fatty acids and ketone bodies can potentially also be explained by accumulation of acetyl-coA. However, these findings need to be validated by future studies.

Ketone bodies are synthesized by the liver and are used as an energy source for the brain, heart and kidney cortex in the absence of sufficient glucose. It has been shown that the diminishing supply of glucose forces the brain and heart to metabolize lactate and ketones as their primary energy sources [26]. Whether decreased utilization of ketone bodies by other organs such as muscle and kidney in order to enhance supplies to the brain during starvation could increase blood levels, is an intriguing possibility.

A study by Huang et al. reported an increase in total phospholipid concentration in addition to individual phospholipid classes in the placental tissue from pre-eclamptic patients [27]. The elevated placental lipid profile in pre-eclampsia was highly associated with the placental dysfunction, as such dysregulation of transport across the placental syncytiotrophoblast and oxidative stress-induced lipid peroxide insult. Preeclampsia is one of the most significant causes of IUGR [28] and further implicates dysregulation of phospholipid metabolism in the development IUGR.

We also identified dysregulation of urea metabolism in IUGR in the cord blood. One might expect lower urea concentrations in neonates and fetuses due to the smaller mass of muscle in IUGR. Several studies have found evidence of impaired urea production in both IUGR neonates and fetuses. [29] The liver plays an important role in urea production. Studies conducted on fetal guinea‐pigs with IUGR generated by unilateral uterine artery ligation found significantly reduced hepatic urea cycle enzymes activity. The urea cycle enzymes are responsible for the metabolism of ammonia which is toxic in elevated concentrations to urea. The ammonia concentration in fetal liver slices were almost 16‐fold higher than in the IUGR fetuses than in controls [30]. Furthermore, a progressive decrease in urea production in the ovine IUGR fetus in late gestation has been demonstrated [31].

Enrichment analysis further identified methionine metabolism to be significantly perturbed in IUGR which is confirmed by previous studies done by MacKay et. al (2012) [32]. Methionine is considered an essential amino acid and one of the key components of one-carbon metabolism that through the ubiquitous methyl donor, s-adenosyl methionine, provides the methyl groups for numerous methyl transferase reactions [33]. Changes to methyl group availability in utero can lead to epigenetic changes manifested by altered DNA methylation and ultimately gene transcription. This has been implicated in "fetal programming", a phenomenon associated with poor nutrition during fetal development that results in low birth weight and disease in later life.

Finally, we found evidence of significant dysregulation in lysine degradation metabolism. Perturbation of this metabolic cascade either by nutrient deficiency, or by nutrient, hormonal and environmental interactions can have a profound impact on the cell function, metabolism, growth and proliferation [34]. This may have a knock-on effect to the growing embryo and subsequent fetus. Human fetuses with IUGR have reduced plasma concentrations of α-aminonitrogen, an overall measure of amino acid content, which appears to be caused by the lowered concentrations of a number of essential amino acids [35]. It has been previously reported that the activity of transporters for essential amino acids in human placentas obtained from IUGR pregnancies was reduced [36] and could relate to the dysregulation in lysine degradation pathway that we observed.

One of the main limitations of this study was the relatively sample size and that our findings were not validated using an independent cohort. However, by applying 10-fold cross validation on the training data set we assured the generalizability of our findings to other data-sets. Also, the inclusion criteria for IUGR was babies was birth weight <10^th^ percentile which does not sufficiently distinguish between constitutional from pathological causes of small fetuses/ newborns. Objective criteria such as subcutaneous fat assessment or body density measurements for the determination of pathological IUGR could have enhanced the insights into the metabolomic basis of growth restriction.

## Conclusion

In the current study we have for the first-time combined data acquired using ^1^H NMR and DI-LS-MS/MS combined with several robust Artificial Intelligence approaches to identify biomarkers for the detection of IUGR as determined by birth weight criterion. High predictive accuracies were achieved. In addition, we provide unique and biologically plausible insights into the metabolic basis of IUGR using actual fetal tissue i.e. cord blood obtained at birth. There appeared to be marked disturbances in liver function which is consistent with evidence from other approaches. We believe that metabolite markers as presented herein could have future clinical utility for the diagnosis of IUGR if they are able to be identified prenatally in maternal serum. Based on these results further work is warranted to validate these findings in a much larger cohort.

## Supporting information

S1 Fig Presenting score plots of principal component analysis (PCA) for outlier detection; controls (A) and IUGR (B) case, respectively.
(TIF)

S1 Table Comparison of demographics and clinical assessments. (DOCX)

S2 Table Optimum *C*-*γ* pairs for each metabolite set and corresponding model performances in terms of accuracies (average and standard deviations) and significance score for 10th percentile. (DOCX)

S3 Table Model performances in terms of accuracies, sensitivities and specificities (average and standard deviations) and significance score when samples grouping was taken place according to 5th %le criteria for each model that provided various panel of metabolites for 5th percentile. (DOCX)

S4 Table Results of univariate analysis comparing concentration of metabolites in serum obtained cord blood of IUGR sufferers and corresponding healthy controls. (DOCX)

## Abbreviations

FGR: fetal growth restriction
IUGR: Intrauterine growth restriction
AI: Artificial Intelligence
^1^ H NMR: proton nuclear magnetic resonance spectroscopy
DI-LC-MS/MS: direct flow injection mass spectrometry with reverse phase liquid chromatography mass spectrometry
AUC: area under the receiver operating curve/are under curve
CI: 349 confidence interval
CV: cross validation
MS: mass spectrometry
PCA: principal component analysis
PLS: partial least squares regression
CFS: Correlation-based feature selection
LVQ: Learning Vector Quantization
MSEA: metabolite set enrichment analysis
