# Supplementary material for: Artificial intelligence and the analysis of multi-platform metabolomics data for the detection of intrauterine growth restriction
Source: PLoS One. 2019 Apr 18;14(4):e0214121. doi: 10.1371/journal.pone.0214121 (PMC6472728; doi:10.1371/journal.pone.0214121)
Supplement: S1 Table — (DOCX) [file pone.0214121.s002.docx]

**S1 Table.** Comparison of demographics and clinical assessments

| ^ϯ^ | **Mean (SD)** | | p-value |
| --- | --- | --- | --- |
|  | IUGR | Controls |  |
| Number | 39 | 39 |  |
| Age | 32.2 (±5.9) | 30.9 (±5.29) | 0.36^+^ |
| Race |  |  |  |
| - White (n) - Black (n) - Asian (n) - Mixed | 29  3  1  3 | 30  4  0  2 | 0.35 ^*^ |
| Gravidity | 3.1 (0.43) | 3.4 (0.52) | 0.74^+^ |
| Prior History of IUGR (n) | 3 | 2 | 0.40 ^δ^ |

^δ^ Chi square test

^+^ t test

* Kruskal-Wallis

^ϯ^ In order to determine growth percentile, we used the growth chart that was developed using our birth weight data which was collected from the William Beaumont Hospital patient population between Jan 1991 – Dec 2005.
