# Supplementary material for: Artificial intelligence and the analysis of multi-platform metabolomics data for the detection of intrauterine growth restriction
Source: PLoS One. 2019 Apr 18;14(4):e0214121. doi: 10.1371/journal.pone.0214121 (PMC6472728; doi:10.1371/journal.pone.0214121)
Supplement: S2 Table — (DOCX) [file pone.0214121.s003.docx]

**S2 Table.** Optimum $C$-$\gamma$ pairs for each metabolite set and corresponding model performances in terms of accuracies (average and standard deviations) and significance score.

|  | $\boldsymbol{C}$ | $\boldsymbol{\gamma}$ | **Accuracy**  **(μ, σ)** | ***p*-value** | ***AUC*** | ***Sensitivity*** | **Specificity** |
| --- | --- | --- | --- | --- | --- | --- | --- |
| **ALL** | 10 | 0.01 | (0.77, 0.16) | 0.0699 | 0.90 | 0.83 | 0.80 |
| **CFS** | 10 | 0.01 | (0.80, 0.15) | 0.00999 | 0.91 | 0.87 | 0.83 |
| **PLS** | 100 | 0.001 | (0.80, 0.14) | 0.00099 | 0.87 | 0.83 | 0.85 |
| **COR-LVQ** | 1 | 0.01 | (0.78, 0.11) | 0.00099 | 0.88 | 0.85 | 0.79 |
| **OL** | 100 | 0.001 | (0.82, 0.15) | 0.00099 | 0.88 | 0.83 | 0.87 |
| **Clinical** | 100 | 0.00001 | (0.54, 0.07) | 1.0 | 0.54 | 0.46 | 0.53 |
| **OL + Clinical** | 1000 | 0.001 | (0.68, 0.27) | 0.00999 | 0.69 | 0.70 | 0.73 |
