# Supplementary material for: Artificial intelligence and the analysis of multi-platform metabolomics data for the detection of intrauterine growth restriction
Source: PLoS One. 2019 Apr 18;14(4):e0214121. doi: 10.1371/journal.pone.0214121 (PMC6472728; doi:10.1371/journal.pone.0214121)
Supplement: S3 Table — (DOCX) [file pone.0214121.s004.docx]

**S3 Table.** Model performances in terms of accuracies, sensitivities and specificities (average and standard deviations) and significance score when samples grouping was taken place according to 5^th^ %le criteria for each model that provided various panel of metabolites.

| **Feature selection schemes** | | | | | |  |
| --- | --- | --- | --- | --- | --- | --- |
| **Metabolic Panel** | **CFS** | **PLS** | **COR-LVQ** | **OL** | |  |
|  | Glu | C2 | C2 | C6..C4.1.DC. | |  |
|  | Alpha AAA | C4 | C6.C4.1.DC. | C2 | |  |
|  | Carnosine | Alpha AAA | C4 | C4 | |  |
|  | C2 | lysoPC.a.C18.2 | 2-Hydroxybutyrate | alpha.AAA | |  |
|  | C4 | PC.aa.C36.4 | PC.aa.C38.6 | lysoPC.a.C20.3 | |  |
|  | C6.C4.1.DC | PC.aa.C38.6 | lysoPC.a.C18.1 | 2-Hydroxybutyrate | |  |
|  | lysoPC.a.C20.3 | lysoPC.a.C16.1 | lysoPC.a.C16.1 |  | |  |
|  | lysoPC.a.C20.4 | C6.C4.1.DC. | lysoPC.a.C18.2 |  | |  |
|  | PC.aa.C36.4 | creatinine | lysoPC.a.C20.3 |  | |  |
|  | PC.aa.C38.5 | lysoPC.a.C18.1 | lysoPC.a.C24.0 |  | |  |
|  | 2-Hydroxybutyrate | lysoPC.a.C20.3 | lysoPC.a.C26.0 |  | |  |
|  | betaine | 2-Hydroxybutyrate | Alpha AAA |  | |  |
|  |  |  | lysoPC.a.C28.1 |  | |  |
|  |  |  | lysoPC.a.C28.1 |  | |  |
|  |  |  | Creatinine |  | |  |
|  | **CFS** | **PLS** | **COR-LVQ** | **OL** | **OL+Clinical data** | |
| **AUC** | 0.93 | 0.87 | 0.88 | 0.94 | 0.93 | |
| **Sensitivity** | 1.00 | 0.94 | 0.92 | 0.90 | 0.89 | |
| **Specificity** | 0.88 | 0.92 | 0.93 | 0.88 | 0.90 | |
| **Significance** | 0.013 | 0.023 | 0.020 | 0.015 | 0.013 | |
