# Supplementary material for: Artificial intelligence and the analysis of multi-platform metabolomics data for the detection of intrauterine growth restriction
Source: PLoS One. 2019 Apr 18;14(4):e0214121. doi: 10.1371/journal.pone.0214121 (PMC6472728; doi:10.1371/journal.pone.0214121)
Supplement: S4 Table — (DOCX) [file pone.0214121.s005.docx]

**S4 Table.** Results of univariate analysis comparing concentration of metabolites in serum obtained cord blood of IUGR sufferers and corresponding healthy controls.

| **Name** | **HMDB IDs** | **Mean (SD) of Controls** | **Mean (SD) of IUGR** | **p-value** | **q-value (FDR)** | **Fold Change** | **0/1** |
| --- | --- | --- | --- | --- | --- | --- | --- |
| Ala | HMDB00161 | 496.308 (76.914) | 526.667 (139.184) | 0.2379 | 0.5802 | -1.06 | Down |
| Asn | HMDB00168 | 43.346 (6.331) | 43.382 (7.435) | 0.9562 (W) | 0.9798 | -1 | Down |
| Asp | HMDB00191 | 19.087 (6.946) | 22.710 (15.163) | 0.5928 (W) | 0.8612 | -1.19 | Down |
| Cit | HMDB00904 | 13.257 (2.464) | 13.943 (3.390) | 0.3104 | 0.6295 | -1.05 | Down |
| Gln | HMDB00641 | 638.718 (63.101) | 638.205 (113.544) | 0.9804 | 0.9888 | 1 | Up |
| Glu | HMDB00148 | 54.231 (18.532) | 68.762 (39.372) | 0.1573 (W) | 0.4587 | -1.27 | Down |
| Gly | HMDB31334 | 292.103 (33.139) | 294.051 (55.252) | 0.8508 | 0.9328 | -1.01 | Down |
| His | HMDB00177 | 135.082 (20.147) | 132.790 (47.482) | 0.1086 (W) | 0.3917 | 1.02 | Up |
| Ile | HMDB00172 | 71.362 (9.749) | 70.405 (11.310) | 0.6903 | 0.8892 | 1.01 | Up |
| **Lys** | **HMDB00182** | **547.282 (63.278)** | **502.436 (100.888)** | **0.0495 (W)** | **0.2385** | **1.09** | **Up** |
| Met | HMDB00696 | 27.810 (4.084) | 29.072 (5.058) | 0.2293 | 0.5718 | -1.05 | Down |
| Orn | HMDB03416 | 90.503 (16.211) | 88.185 (21.729) | 0.5949 | 0.8612 | 1.03 | Up |
| Phe | HMDB00159 | 76.105 (8.680) | 83.959 (21.296) | 0.0325 (W) | 0.1816 | -1.1 | Down |
| Pro | HMDB00162 | 128.946 (19.855) | 133.564 (24.448) | 0.3627 | 0.6953 | -1.04 | Down |
| Ser | HMDB00187 | 167.385 (21.806) | 170.333 (31.418) | 0.6317 | 0.8835 | -1.02 | Down |
| **Thr** | **HMDB06083** | **280.513 (49.242)** | **264.487 (82.504)** | **0.0400 (W)** | **0.0207** | **1.06** | **Up** |
| Trp | HMDB00929 | 83.026 (8.114) | 78.913 (14.554) | 0.2079 (W) | 0.5449 | 1.05 | Up |
| Tyr | HMDB00158 | 69.336 (11.795) | 66.808 (11.878) | 0.9363 (W) | 0.9773 | 1.04 | Up |
| Val | HMDB00883 | 247.590 (28.370) | 243.590 (35.550) | 0.5845 | 0.8612 | 1.02 | Up |
| Ac.Orn | HMDB03357 | 0.101 (0.168) | 0.189 (0.371) | 0.2437 (W) | 0.5867 | -1.86 | Down |
| ADMA | HMDB01539 | 1.269 (0.217) | 1.230 (0.247) | 0.7796 (W) | 0.9328 | 1.03 | Up |
| alpha.AAA | HMDB00510 | 0.841 (0.119) | 0.974 (0.342) | 0.0531 (W) | 0.25 | -1.16 | Down |
| c4.OH.Pro | HMDB06055 | 0.167 (0.099) | 0.132 (0.102) | 0.0918 (W) | 0.3585 | 1.27 | Up |
| Carnosine | HMDB00033 | 0.976 (0.524) | 0.836 (0.489) | 0.4240 (W) | 0.7632 | 1.17 | Up |
| **Creatinine** | **HMDB00562** | **43.131 (17.101)** | **62.069 (23.726)** | **0.0001 (W)** | **0.0054** | **-1.44** | **Down** |
| **DOPA** | **HMDB00073** | **0.251 (0.012)** | **0.240 (0.014)** | **0.0009** | **0.0253** | **1.04** | **Up** |
| Dopamine | HMDB00073 | 0.179 (0.099) | 0.168 (0.106) | 0.8519 (W) | 0.9328 | 1.07 | Up |
| Histamine | HMDB00870 | 0.213 (0.005) | 0.212 (0.004) | 0.2731 (W) | 0.6295 | 1.01 | Up |
| **Kynurenine** | **HMDB00684** | **5.640 (1.039)** | **4.752 (1.188)** | **0.0010 (W)** | **0.0253** | **1.19** | **Up** |
| Met.SO | HMDB02005 | 0.323 (0.085) | 0.314 (0.134) | 0.7094 | 0.8892 | 1.03 | Up |
| Putrescine | HMDB01414 | 0.164 (0.074) | 1.550 (8.452) | 0.0258 (W) | 0.162 | -9.47 | Down |
| Sarcosine | HMDB00271 | 21.608 (3.596) | 22.928 (6.793) | 0.7950 (W) | 0.9328 | -1.06 | Down |
| Serotonin | HMDB00259 | 0.250 (0.104) | 0.274 (0.182) | 0.5757 (W) | 0.8612 | -1.1 | Down |
| Spermidine | HMDB01256 | 0.312 (0.296) | 0.371 (0.215) | 0.1459 (W) | 0.4549 | -1.19 | Down |
| t4.OH.Pro | HMDB59659 | 17.746 (3.864) | 19.246 (5.513) | 0.1837 (W) | 0.5004 | -1.08 | Down |
| Taurine | HMDB00251 | 185.026 (55.102) | 153.790 (74.626) | 0.0101 (W) | 0.1072 | 1.2 | Up |
| SDMA | HMDB03334 | 1.672 (0.376) | 1.708 (0.519) | 0.7302 (W) | 0.9051 | -1.02 | Down |
| **C0** | **HMDB00062** | **15.787 (3.543)** | **19.157 (5.427)** | **0.0006 (W)** | **0.021** | **-1.21** | **Down** |
| C10 | HMDB00651 | 0.070 (0.020) | 0.083 (0.036) | 0.2149 (W) | 0.5493 | -1.19 | Down |
| **C10:1** | **HMDB00651** | **0.290 (0.087)** | **0.385 (0.165)** | **0.0011 (W)** | **0.0253** | **-1.33** | **Down** |
| C10:2 | HMDB06469 | 0.043 (0.019) | 0.046 (0.022) | 0.6170 (W) | 0.8752 | -1.07 | Down |
| C12 | HMDB02250 | 0.040 (0.007) | 0.051 (0.026) | 0.0256 (W) | 0.162 | -1.27 | Down |
| C12-DC | HMDB13327 | 0.045 (0.017) | 0.045 (0.023) | 0.9202 (W) | 0.9768 | -1.01 | Down |
| **C12:1** | **---------------** | **0.256 (0.095)** | **0.333 (0.142)** | **0.0017 (W)** | **0.0312** | **-1.3** | **Down** |
| C14 | HMDB05066 | 0.026 (0.004) | 0.032 (0.016) | 0.3132 (W) | 0.6295 | -1.23 | Down |
| C14:1 | HMDB02014 | 0.027 (0.009) | 0.033 (0.017) | 0.2982 (W) | 0.6295 | -1.24 | Down |
| C14:1-OH | HMDB13330 | 0.008 (0.003) | 0.010 (0.005) | 0.3124 (W) | 0.6295 | -1.19 | Down |
| C14:2 | HMDB13331 | 0.012 (0.005) | 0.018 (0.014) | 0.0274 (W) | 0.167 | -1.53 | Down |
| C14:2-OH | ---------------- | 0.007 (0.003) | 0.007 (0.004) | 0.6534 (W) | 0.8892 | -1.08 | Down |
| C16 | HMDB00222 | 0.071 (0.018) | 0.085 (0.035) | 0.1176 (W) | 0.4125 | -1.2 | Down |
| C16:OH | HMDB13336 | 0.010 (0.006) | 0.010 (0.009) | 0.9187 (W) | 0.9768 | -1.06 | Down |
| **C16:1** | **HMDB13207** | **0.013 (0.004)** | **0.021 (0.015)** | **0.0110 (W)** | **0.1083** | **-1.55** | **Down** |
| C16:1-OH | HMDB13333 | 0.008 (0.003) | 0.009 (0.004) | 0.6063 (W) | 0.8716 | -1.08 | Down |
| C16:2 | HMDB13334 | 0.006 (0.003) | 0.009 (0.004) | 0.0236 (W) | 0.1576 | -1.36 | Down |
| C16:2-OH | HMDB13335 | 0.011 (0.003) | 0.014 (0.004) | 0.0064 (W) | 0.0886 | -1.22 | Down |
| C18 | HMDB00848 | 0.022 (0.008) | 0.022 (0.008) | 0.9361 (W) | 0.9773 | -1.02 | Down |
| C18:1 | HMDB05065 | 0.034 (0.009) | 0.044 (0.023) | 0.0936 (W) | 0.3589 | -1.29 | Down |
| C18:1-OH | HMDB13339 | 0.005 (0.003) | 0.006 (0.004) | 0.5192 (W) | 0.8268 | -1.22 | Down |
| C18:2 | HMDB06461 | 0.023 (0.006) | 0.029 (0.014) | 0.0314 (W) | 0.1816 | -1.25 | Down |
| **C2** | **HMDB00201** | **2.404 (0.613)** | **3.767 (2.005)** | **0.0032 (W)** | **0.051** | **-1.57** | **Down** |
| C3 | HMDB00824 | 0.290 (0.129) | 0.340 (0.128) | 0.0104 (W) | 0.1072 | -1.18 | Down |
| C3-OH | HMDB13125 | 0.013 (0.007) | 0.012 (0.010) | 0.3127 (W) | 0.6295 | 1.06 | Up |
| C3:1 | HMDB13124 | 0.016 (0.008) | 0.019 (0.012) | 0.7588 (W) | 0.9252 | -1.14 | Down |
| **C4** | **HMDB02013** | **0.087 (0.022)** | **0.117 (0.049)** | **0.0013 (W)** | **0.0275** | **-1.35** | **Down** |
| **C3-DC (C4-OH)** | **HMDB02095** | **0.055 (0.021)** | **0.075 (0.041)** | **0.0128 (W)** | **0.1107** | **-1.37** | **Down** |
| C4:1 | HMDB13126 | 0.028 (0.010) | 0.031 (0.017) | 0.5025 (W) | 0.8135 | -1.07 | Down |
| C5 | HMDB13128 | 0.129 (0.048) | 0.143 (0.083) | 0.9841 (W) | 0.9888 | -1.11 | Down |
| C5-M-DC | HMDB00552 | 0.031 (0.016) | 0.032 (0.022) | 0.7074 (W) | 0.8892 | -1.05 | Down |
| C5-OH (C3-DC-M) | HMDB13133 | 0.030 (0.012) | 0.033 (0.012) | 0.1437 (W) | 0.4549 | -1.09 | Down |
| C5:1 | HMDB02366 | 0.024 (0.014) | 0.033 (0.023) | 0.1330 (W) | 0.437 | -1.35 | Down |
| C5:1-DC | HMDB13129 | 0.020 (0.011) | 0.021 (0.015) | 0.4286 (W) | 0.7648 | -1.03 | Down |
| C6 (C4:1-DC) | HMDB00705 | 0.037 (0.010) | 0.055 (0.033) | 0.0102 (W) | 0.1072 | -1.49 | Down |
| C5-DC (C6-OH) | HMDB13131 | 0.018 (0.011) | 0.023 (0.016) | 0.1288 (W) | 0.437 | -1.26 | Down |
| C6:1 | HMDB13161 | 0.024 (0.017) | 0.024 (0.013) | 0.6447 (W) | 0.8892 | -1 | Down |
| C7-DC | HMDB13328 | 0.016 (0.008) | 0.018 (0.011) | 0.6992 (W) | 0.8892 | -1.11 | Down |
| C8 | HMDB00791 | 0.086 (0.040) | 0.096 (0.053) | 0.1601 (W) | 0.4604 | -1.12 | Down |
| C9 | HMDB13288 | 0.016 (0.006) | 0.019 (0.010) | 0.3057 (W) | 0.6295 | -1.19 | Down |
| lysoPC a C14:0 | HMDB10379 | 2.199 (0.229) | 2.092 (0.171) | 0.0188 (W) | 0.1335 | 1.05 | Up |
| lysoPC a C16:0 | HMDB10382 | 37.964 (5.660) | 34.762 (7.168) | 0.0316 | 0.1816 | 1.09 | Up |
| lysoPC a C16:1 | HMDB10383 | 3.082 (0.622) | 2.306 (0.751) | < 0.0001 | 0 | 1.34 | Up |
| lysoPC a C17:0 | HMDB12108 | 0.419 (0.086) | 0.406 (0.119) | 0.3605 (W) | 0.6953 | 1.03 | Up |
| lysoPC a C18:0 | HMDB10384 | 7.703 (1.208) | 7.204 (1.706) | 0.0563 (W) | 0.2589 | 1.07 | Up |
| **lysoPC a C18:1** | **HMDB02815** | **8.639 (1.758)** | **6.847 (2.062)** | **< 0.0001** | **0.0054** | **1.26** | **Up** |
| lysoPC a C18:2 | HMDB10386 | 9.508 (2.464) | 7.339 (2.623) | < 0.0001 (W) | 0.0054 | 1.3 | Up |
| **lysoPC a C20:3** | **HMDB10394** | **2.362 (0.677)** | **1.770 (0.760)** | **0.0001 (W)** | **0.0054** | **1.33** | **Up** |
| lysoPC a C20:4 | HMDB10395 | 9.619 (2.461) | 8.252 (3.168) | 0.0120 (W) | 0.1107 | 1.17 | Up |
| lysoPC a C24:0 | HMDB10405 | 0.081 (0.021) | 0.090 (0.021) | 0.0145 (W) | 0.1116 | -1.12 | Down |
| lysoPC a C26:0 | HMDB29205 | 0.111 (0.029) | 0.125 (0.029) | 0.0134 (W) | 0.1107 | -1.13 | Down |
| lysoPC a C26:1 | HMDB29220 | 0.053 (0.013) | 0.058 (0.011) | 0.0680 (W) | 0.2873 | -1.09 | Down |
| lysoPC a C28:0 | HMDB29206 | 0.101 (0.022) | 0.100 (0.021) | 0.915 | 0.9768 | 1.01 | Up |
| lysoPC a C28:1 | HMDB29221 | 0.081 (0.014) | 0.094 (0.023) | 0.0095 (W) | 0.1072 | -1.16 | Down |
| **PC.aa.C24.0** | **----------------** | **0.079 (0.013)** | **0.095 (0.025)** | **0.0018 (W)** | **0.0312** | **-1.2** | **Down** |
| **PC.aa.C26.0** | **----------------** | **0.468 (0.054)** | **0.482 (0.055)** | **0.0623 (W)** | **0.02774** | **-1.3** | **Down** |
| **PC aa C28:1** | **HMDB07899** | **0.683 (0.167)** | **0.740 (0.214)** | **0.02760 (W)** | **0.06295** | **-1.8** | **Down** |
| **PC aa C30:0** | **HMDB07869** | **2.777 (0.707)** | **2.787 (0.725)** | **0.04522** | **0.09798** | **-1.7** | **Down** |
| **PC aa C32:0** | **HMDB07871** | **14.386 (3.884)** | **15.686 (4.055)** | **0.01632 (W)** | **0.04628** | **-1.9** | **Down** |
| **PC aa C32:1** | **HMDB07969** | **11.350 (3.650)** | **11.210 (9.120)** | **0.08484 (W)** | **0.03517** | **-1.5** | **Down** |
| PC aa C32:2 | HMDB07874 | 0.116 (0.030) | 0.122 (0.054) | 0.6856 (W) | 0.8892 | -1.05 | Down |
| PC aa C34:1 | HMDB07972 | 99.382 (23.155) | 103.210 (45.673) | 0.5894 (W) | 0.8612 | -1.04 | Down |
| **PC aa C34:2** | **HMDB07973** | **82.941 (21.350)** | **94.526 (49.953)** | **0.01973 (W)** | **0.05305** | **-1.14** | **Down** |
| PC aa C34:3 | HMDB07974 | 1.707 (0.443) | 2.191 (3.208) | 0.8338 (W) | 0.9328 | -1.28 | Down |
| PC aa C34:4 | HMDB07883 | 0.251 (0.061) | 0.298 (0.352) | 0.2940 (W) | 0.6295 | -1.18 | Down |
| PC aa C36:0 | HMDB08036 | 2.431 (0.419) | 2.515 (0.559) | 0.4517 | 0.7792 | -1.03 | Down |
| **PC aa C36:1** | **HMDB08037** | **18.372 (3.803)** | **17.621 (4.447)** | **0.1473 (W)** | **0.04549** | **1.04** | **Up** |
| PC aa C36:2 | HMDB00593 | 43.251 (10.360) | 46.790 (25.056) | 0.6279 (W) | 0.8835 | -1.08 | Down |
| **PC aa C36:3** | **HMDB07980** | **46.405 (10.969)** | **46.313 (23.581)** | **0.3080 (W)** | **0.06295** | **1** | **Up** |
| **PC aa C36:4** | **HMDB08138** | **124.072 (25.425)** | **144.192 (40.452)** | **0.0337 (W)** | **0.01834** | **-1.16** | **Down** |
| PC aa C36:5 | HMDB07984 | 2.248 (0.604) | 2.540 (1.701) | 0.8651 (W) | 0.9328 | -1.13 | Down |
| **PC aa C36:6** | **HMDB07892** | **0.096 (0.026)** | **0.099 (0.080)** | **0.0630 (W)** | **0.02774** | **-1.03** | **Down** |
| PC aa C38:0 | HMDB07893 | 1.220 (0.219) | 1.273 (0.296) | 0.3763 | 0.7145 | -1.04 | Down |
| **PC aa C38:3** | **HMDB08046** | **32.467 (6.729)** | **29.467 (5.661)** | **0.0364** | **0.0193** | **1.1** | **Up** |
| **PC aa C38:4** | **HMDB08048** | **87.431 (17.461)** | **98.015 (20.056)** | **0.0194 (W)** | **0.01335** | **-1.12** | **Down** |
| **PC aa C38:5** | **HMDB07989** | **14.909 (3.629)** | **16.438 (6.243)** | **0.3578 (W)** | **0.06953** | **-1.1** | **Down** |
| PC aa C38:6 | HMDB07991 | 41.590 (9.179) | 44.579 (13.429) | 0.5826 (W) | 0.8612 | -1.07 | Down |
| PC aa C40:1 | HMDB07993 | 0.208 (0.027) | 0.210 (0.035) | 0.9801 (W) | 0.9888 | -1.01 | Down |
| PC aa C40:2 | HMDB08276 | 0.096 (0.037) | 0.089 (0.036) | 0.5030 (W) | 0.8135 | 1.08 | Up |
| PC aa C40:3 | HMDB08119 | 0.406 (0.083) | 0.406 (0.099) | 0.9912 | 0.9912 | -1 | Down |
| **PC aa C40:4** | **HMDB08054** | **3.227 (0.847)** | **3.415 (0.691)** | **0.01076 (W)** | **0.03917** | **-1.6** | **Down** |
| PC aa C40:5 | HMDB08055 | 4.583 (1.499) | 4.516 (1.279) | 0.8143 (W) | 0.9328 | 1.01 | Up |
| **PC aa C40:6** | **HMDB08057** | **17.878 (4.553)** | **18.321 (5.494)** | **0.06991** | **0.08892** | **-1.2** | **Down** |
| PC aa C42:0 | HMDB08058 | 0.432 (0.107) | 0.410 (0.130) | 0.3399 (W) | 0.67 | 1.05 | Up |
| PC aa C42:1 | HMDB08059 | 0.243 (0.053) | 0.242 (0.083) | 0.5289 (W) | 0.8295 | 1 | Up |
| **PC aa C42:2** | **HMDB08284** | **0.114 (0.023)** | **0.0117 (0.024)** | **0.08298 (W)** | **0.9328** | **-1.2** | **Down** |
| **PC aa C42:4** | **HMDB08285** | **0.233 (0.052)** | **0.0268 (0.060)** | **0.009** | **0.1072** | **-1.5** | **Down** |
| **PC aa C42:5** | **HMDB08287** | **0.229 (0.053)** | **0.0254 (0.062)** | **0.0445 (W)** | **0.2248** | **-1.4** | **Down** |
| **PC aa C42:6** | **HMDB08288** | **0.259 (0.048)** | **0.0261 (0.062)** | **0.03783 (W)** | **0.8892** | **-1.3** | **Down** |
| PC ae C30:0 | HMDB13341 | 0.221 (0.050) | 0.210 (0.044) | 0.3164 | 0.6298 | 1.05 | Up |
| PC ae C30:1 | HMDB13402 | 0.024 (0.019) | 0.022 (0.017) | 0.7601 (W) | 0.9252 | 1.1 | Up |
| **PC ae C30:2** | **HMDB13410** | **0.030 (0.006)** | **0.032 (0.007)** | **0.03839 (W)** | **0.7225** | **-1.7** | **Down** |
| PC ae C32:1 | HMDB13404 | 2.110 (0.624) | 2.120 (0.554) | 0.8066 (W) | 0.9328 | -1 | Down |
| PC ae C32:2 | HMDB13411 | 0.421 (0.128) | 0.419 (0.107) | 0.8612 (W) | 0.9328 | 1 | Up |
| PC ae C34:0 | HMDB13405 | 0.883 (0.220) | 0.929 (0.218) | 0.4656 (W) | 0.7966 | -1.05 | Down |
| PC ae C34:1 | HMDB13426 | 3.961 (0.941) | 4.024 (1.463) | 0.6893 (W) | 0.8892 | -1.02 | Down |
| PC ae C34:2 | HMDB11151 | 2.783 (0.668) | 2.913 (1.011) | 0.7643 (W) | 0.9252 | -1.05 | Down |
| PC ae C34:3 | HMDB13413 | 0.946 (0.223) | 1.018 (0.405) | 0.8143 (W) | 0.9328 | -1.08 | Down |
| **PC ae C36:0** | **HMDB13406** | **0.504 (0.103)** | **0.531 (0.121)** | **0.02779** | **0.0295** | **-1.06** | **Down** |
| PC ae C36:1 | HMDB13414 | 5.392 (1.042) | 5.992 (2.991) | 0.5554 (W) | 0.8517 | -1.11 | Down |
| PC ae C36:2 | HMDB13418 | 2.083 (0.457) | 2.473 (2.031) | 0.6385 (W) | 0.8871 | -1.19 | Down |
| **PC ae C36:3** | **HMDB13429** | **1.404 (0.349)** | **1.361 (0.634)** | **0.01573 (W)** | **0.04587** | **1.03** | **Down** |
| PC ae C36:4 | HMDB13407 | 8.351 (1.678) | 8.630 (2.400) | 0.9403 (W) | 0.9773 | -1.03 | Down |
| **PC ae C36:5** | **HMDB13415** | **5.051 (1.258)** | **5.569 (1.461)** | **0.01544 (W)** | **0.04587** | **-1.4** | **Down** |
| PC ae C38:0 | HMDB13408 | 0.546 (0.120) | 0.575 (0.194) | 0.5521 (W) | 0.8517 | -1.05 | Down |
| PC ae C38:2 | HMDB13431 | 0.136 (0.166) | 0.130 (0.193) | 0.4477 (W) | 0.7787 | 1.04 | Up |
| PC ae C38:3 | HMDB13439 | 2.757 (0.641) | 2.717 (0.856) | 0.4096 (W) | 0.7556 | 1.01 | Up |
| **PC ae C38:4** | **HMDB13420** | **6.205 (1.175)** | **6.727 (1.552)** | **0.01432 (W)** | **0.04549** | **-1.8** | **Down** |
| PC ae C38:5 | HMDB13432 | 5.192 (1.036) | 5.536 (1.290) | 0.4125 (W) | 0.7556 | -1.07 | Down |
| **PC ae C38:6** | **HMDB13409** | **1.912 (0.419)** | **2.051 (0.531)** | **0.0204** | **0.05414** | **-1.27** | **Down** |
| PC ae C40:1 | HMDB13433 | 0.607 (0.163) | 0.624 (0.245) | 0.7004 (W) | 0.8892 | -1.03 | Down |
| PC ae C40:2 | HMDB13437 | 0.646 (0.150) | 0.642 (0.165) | 0.7116 (W) | 0.8892 | 1.01 | Up |
| PC ae C40:3 | HMDB13445 | 0.830 (0.178) | 0.849 (0.185) | 0.8651 (W) | 0.9328 | -1.02 | Down |
| PC ae C40:4 | HMDB13442 | 1.414 (0.315) | 1.444 (0.333) | 0.8376 (W) | 0.9328 | -1.02 | Down |
| PC ae C40:5 | HMDB13444 | 1.611 (0.335) | 1.638 (0.336) | 0.6673 (W) | 0.8892 | -1.02 | Down |
| PC ae C40:6 | HMDB13422 | 1.474 (0.331) | 1.580 (0.408) | 0.2246 (W) | 0.567 | -1.07 | Down |
| PC ae C42:0 | HMDB13443 | 0.672 (0.050) | 0.690 (0.071) | 0.4068 (W) | 0.7556 | -1.03 | Down |
| **PC ae C42:1** | **HMDB13434** | **0.320 (0.054)** | **0.0339 (0.0068)** | **0.02651 (W)** | **0.06295** | **-1.6** | **Down** |
| PC ae C42:2 | HMDB13438 | 0.191 (0.053) | 0.198 (0.048) | 0.9442 (W) | 0.9773 | -1.04 | Down |
| PC ae C42:3 | HMDB13458 | 0.280 (0.060) | 0.268 (0.064) | 0.4179 | 0.7588 | 1.04 | Up |
| PC ae C42:4 | HMDB13448 | 0.348 (0.087) | 0.334 (0.080) | 0.4753 | 0.8065 | 1.04 | Up |
| PC ae C42:5 | HMDB13451 | 0.926 (0.179) | 0.874 (0.182) | 0.2121 | 0.5487 | 1.06 | Up |
| PC ae C44:3 | HMDB13449 | 0.084 (0.014) | 0.083 (0.014) | 0.8142 (W) | 0.9328 | 1.01 | Up |
| PC ae C44:4 | HMDB13453 | 0.218 (0.053) | 0.212 (0.041) | 0.8182 (W) | 0.9328 | 1.03 | Up |
| **PC ae C44:5** | **HMDB13456** | **0.615 (0.159)** | **0.549 (0.157)** | **0.0666** | **0.02873** | **1.2** | **Up** |
| PC ae C44:6 | HMDB13450 | 0.537 (0.130) | 0.508 (0.160) | 0.5290 (W) | 0.8295 | 1.06 | Up |
| SM (OH) C14:1 | HMDB13462 | 1.711 (0.382) | 1.900 (0.526) | 0.1529 (W) | 0.4587 | -1.11 | Down |
| SM (OH) C16:1 | HMDB13463 | 1.430 (0.328) | 1.551 (0.350) | 0.1098 (W) | 0.3917 | -1.08 | Down |
| SM (OH) C22:1 | HMDB13466 | 2.721 (0.621) | 2.882 (0.758) | 0.4999 (W) | 0.8135 | -1.06 | Down |
| SM (OH) C22:2 | HMDB13467 | 2.523 (0.646) | 2.709 (0.737) | 0.3128 (W) | 0.6295 | -1.07 | Down |
| SM (OH) C24:1 | HMDB13469 | 0.662 (0.153) | 0.679 (0.149) | 0.5689 (W) | 0.8612 | -1.03 | Down |
| SM C16:0 | HMDB10169 | 48.038 (9.445) | 50.762 (10.744) | 0.2382 | 0.5802 | -1.06 | Down |
| SM C16:1 | HMDB13464 | 9.491 (2.097) | 9.507 (2.167) | 0.973 | 0.9888 | -1 | Down |
| SM C18:0 | HMDB01348 | 15.346 (3.057) | 15.464 (3.060) | 0.8652 | 0.9328 | -1.01 | Down |
| SM C18:1 | HMDB12101 | 11.989 (2.868) | 11.825 (2.749) | 0.8494 (W) | 0.9328 | 1.01 | Up |
| SM C20:2 | HMDB13465 | 0.306 (0.089) | 0.332 (0.120) | 0.4357 (W) | 0.7695 | -1.08 | Down |
| SM C24:0 | HMDB11697 | 11.816 (1.939) | 11.780 (2.194) | 0.9387 | 0.9773 | 1 | Up |
| SM C24:1 | HMDB12107 | 27.869 (4.976) | 28.754 (5.979) | 0.4798 | 0.8074 | -1.03 | Down |
| SM C26:0 | HMDB11698 | 0.135 (0.033) | 0.145 (0.032) | 0.1668 | 0.4665 | -1.08 | Down |
| SM C26:1 | HMDB13461 | 0.339 (0.071) | 0.346 (0.085) | 0.7023 | 0.8892 | -1.02 | Down |
| Urea | HMDB00294 | 232.907 (257.057) | 191.310 (104.128) | 0.5095 (W) | 0.8176 | 1.22 | Up |
| **Arg** | **HMDB00517** | **50.654 (17.253)** | **43.895 (27.368)** | **0.0777 (W)** | **0.03218** | **1.5** | **Up** |
| Leucine | HMDB00687 | 53.421 (13.464) | 55.733 (18.158) | 0.1214 (W) | 0.4187 | -1.04 | Down |
| **2-Hydroxybutyrate** | **HMDB00008** | **22.285 (12.615)** | **28.175 (17.752)** | **0.0812 (W)** | **0.03295** | **-1.26** | **Down** |
| **3-Hydroxybutyrate** | **HMDB00357** | **56.373 (96.577)** | **89.659 (188.264)** | **0.04968 (W)** | **0.08135** | **-1.59** | **Down** |
| **Acetate** | **HMDB00042** | **23.415 (7.538)** | **26.603 (15.933)** | **0.05454 (W)** | **0.0689** | **-1.14** | **Down** |
| **Acetoacetate** | **HMDB00060** | **33.028 (43.789)** | **41.597 (80.847)** | **0.0338 (W)** | **0.0532** | **-1.26** | **Down** |
| Acetone | HMDB01659 | 13.369 (11.664) | 12.077 (7.941) | 0.6783 (W) | 0.8892 | 1.11 | Up |
| Betaine | HMDB00043 | 31.813 (11.032) | 37.767 (19.794) | 0.1061 | 0.3917 | -1.19 | Down |
| Carnitine | HMDB00062 | 13.123 (4.618) | 16.572 (12.815) | 0.2962 (W) | 0.6295 | -1.26 | Down |
| **Choline** | **HMDB00097** | **22.210 (6.645)** | **30.482 (22.697)** | **0.0478 (W)** | **0.0235** | **-1.37** | **Down** |
| **Creatine** | **HMDB00064** | **26.154 (9.473)** | **34.231 (17.335)** | **0.0133** | **0.1107** | **-1.31** | **Down** |
| Dimethyl.sulfone | HMDB04983 | 3.751 (1.658) | 5.044 (6.357) | 0.2826 (W) | 0.6295 | -1.34 | Down |
| Ethanol | HMDB00108 | 50.295 (134.255) | 65.808 (199.739) | 0.1821 (W) | 0.5004 | -1.31 | Down |
| **Formate** | **HMDB00142** | **27.590 (6.873)** | **30.272 (11.350)** | **0.0145 (W)** | **0.1116** | **-1.1** | **Down** |
| Glucose | HMDB00122 | 2238.174 (536.547) | 2326.103 (832.805) | 0.4926 (W) | 0.8135 | -1.04 | Down |
| Glycerol | HMDB00131 | 554.295 (284.013) | 474.833 (177.022) | 0.7355 (W) | 0.9062 | 1.17 | Up |
| Hypoxanthine | HMDB00157 | 7.856 (2.412) | 8.649 (5.115) | 0.6600 (W) | 0.8892 | -1.1 | Down |
| Isobutyrate | HMDB01873 | 4.510 (1.598) | 5.364 (2.228) | 0.0169 (W) | 0.1246 | -1.19 | Down |
| Isopropanol | HMDB00863 | 5.638 (5.021) | 6.846 (7.516) | 0.8572 (W) | 0.9328 | -1.21 | Down |
| Lactate | HMDB00517 | 2001.569 (704.887) | 2306.577 (1368.468) | 0.7131 (W) | 0.8892 | -1.15 | Down |
| Malonate | HMDB00691 | 11.890 (7.035) | 18.349 (26.779) | 0.1312 (W) | 0.437 | -1.54 | Down |
| Methanol | HMDB01875 | 41.138 (31.307) | 57.933 (58.903) | 0.6173 (W) | 0.8752 | -1.41 | Down |
| Propylene.glycol | HMDB41605 | 33.287 (21.548) | 34.233 (31.726) | 0.4386 (W) | 0.7695 | -1.03 | Down |
| Pyruvate | HMDB00243 | 79.508 (33.494) | 82.872 (59.464) | 0.5894 (W) | 0.8612 | -1.04 | Down |
| Succinate | HMDB00254 | 11.305 (5.739) | 13.736 (8.272) | 0.3057 (W) | 0.6295 | -1.22 | Down |
| Methylhistidine | HMDB00479 | 100.882 (24.513) | 96.449 (38.901) | 0.3057 (W) | 0.6295 | 1.05 | Up |
